# Supplementary material for: Effect of Mifepristone on Migration and Proliferation of Oral Cancer Cells
Source: Int J Mol Sci. 2024 Aug 12;25(16):8777. doi: 10.3390/ijms25168777 (PMC11354386; doi:10.3390/ijms25168777)
Supplement: Supplementary file 1 [file ijms-25-08777-s001.zip › ijms-3072780-supplementary.pdf]

Supplementary Material

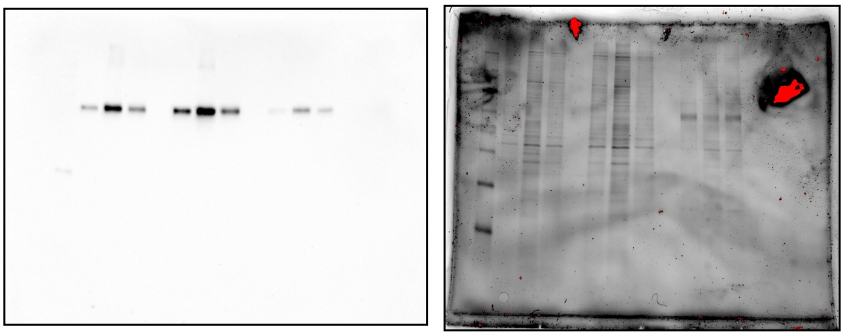

Figure S1: Full blot and gel for glucocorticoid receptor expression in TYS, SAS-H1, and HaCaT cell lines.

Key. Lane 1 = Magic marker, lane 2 = HaCaT SFM 24 h, lane 3 = TYS SFM 24 h, lane 4 = SAS-H1 SFM 24 h, lane 5 = empty, lane 6 = HaCaT SFM 48 h, lane 7 = TYS SFM 48 h, lane 8 = SAS-H1 SF 48 h, lane 9 = empty, lane 10 = HaCaT baseline 24 h, lane 11 = TYS baseline 24 h, and lane 12 = SAS-H1 baseline (Shown in Figure 4.1)

HaCaT Cell line

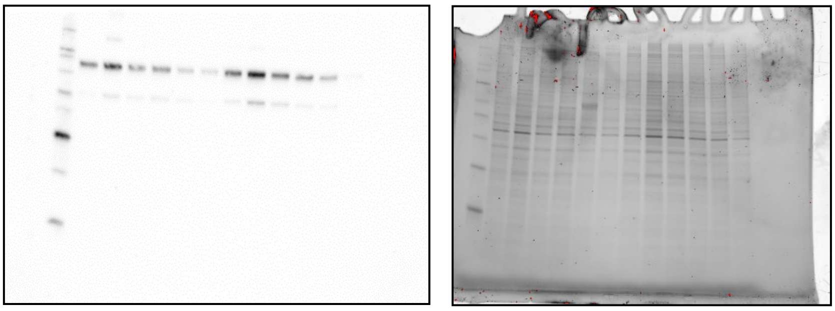

TYS Cell line

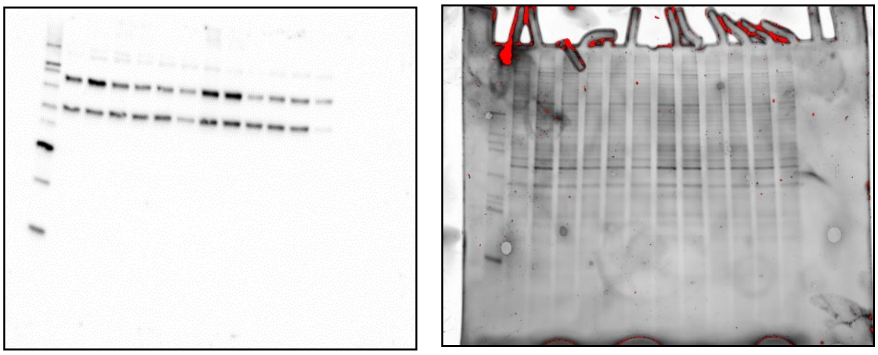

SAS-H1

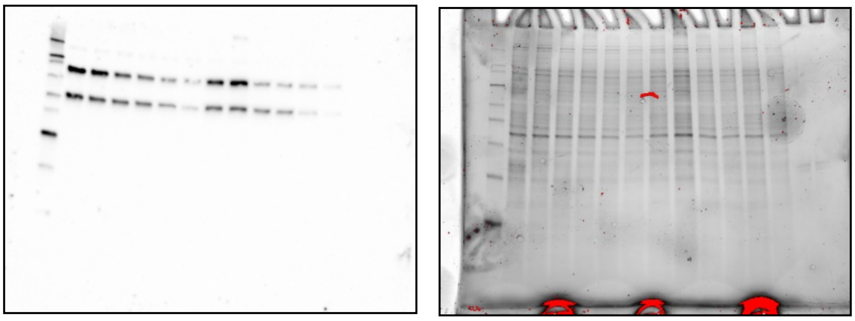

Figure S2: Full blot image and total protein gel for the expression of glucocorticoid receptor and phosphorylated Akt at residue T308 in response to mifepristone in HaCaT, TYS, and SAS-H1 cells. **Key:** Lane 1: magic marker, lane 2: SFM 24 h, lane 3: EGF 24 h, lane 4: Mif 1μM 24 h, lane 5: Mif 5μM 24 h, lane 6: Mif 10 μM 24 h, lane 7: Mif 20 μM 24 h, lane 8: SFM 48 h, lane 9: EGF 48 h, lane 10: Mif 1μM 48 h, lane 11: Mif 5μM 48 h, lane 12: Mif 10μM 48 h, and lane 13: Mif 20μM 48 h.

HaCaT

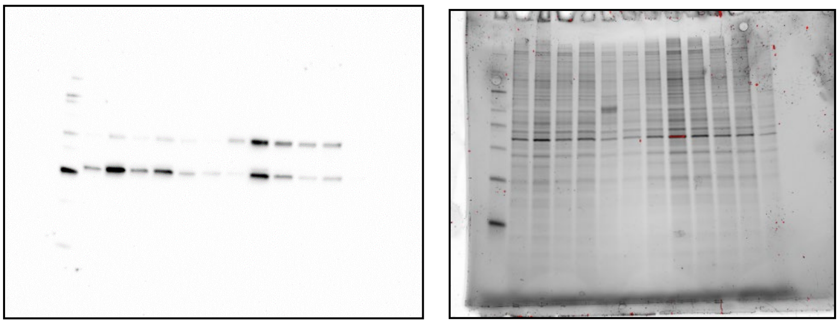

SAS-H1

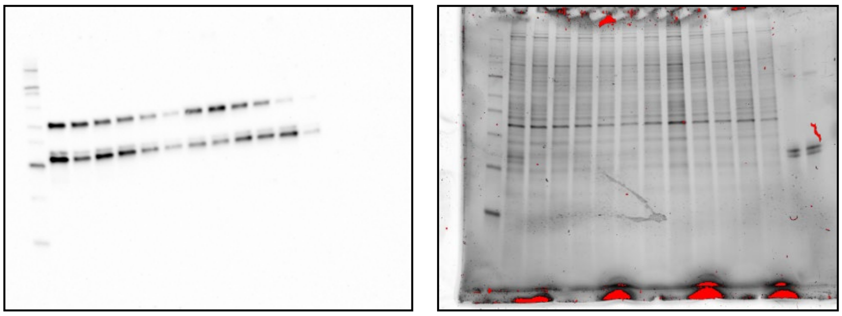

Figure S3: Full blot image and total protein gel for phosphorylated Akt at residue T473 and phospho 44/42 MAPK in response to mifepristone in HaCaT, TYS, and SAS-H1 cells. **Key:** Lane 1: magic marker, lane 2: SFM 24 h, lane 3: EGF 24 h, lane 4: Mif 1μM 24 h, lane 5: Mif 5μM 24 h, lane 6: Mif 10 μM 24 h, lane 7: Mif 20 μM 24 h, lane 8: SFM 48 h, lane 9: EGF 48 h, lane 10: Mif 1μM 48 h, lane 11: Mif 5μM 48 h, lane 12: Mif 10μM 48 h, and lane 13: Mif 20μM 48 h.

HaCaT

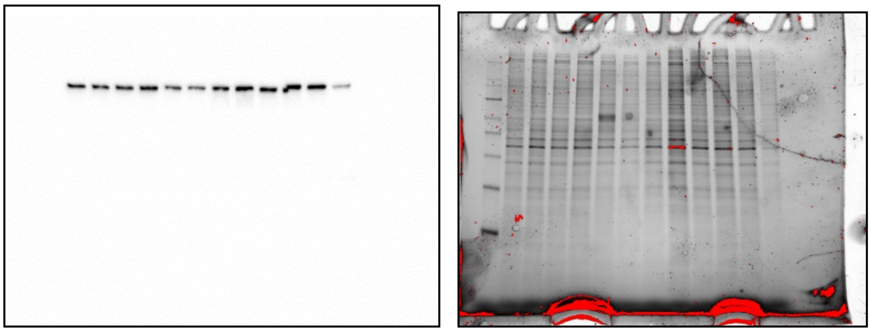

SAS-H1

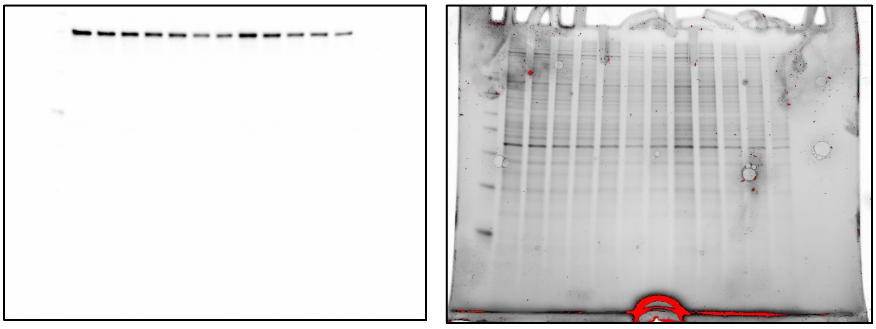

TYS

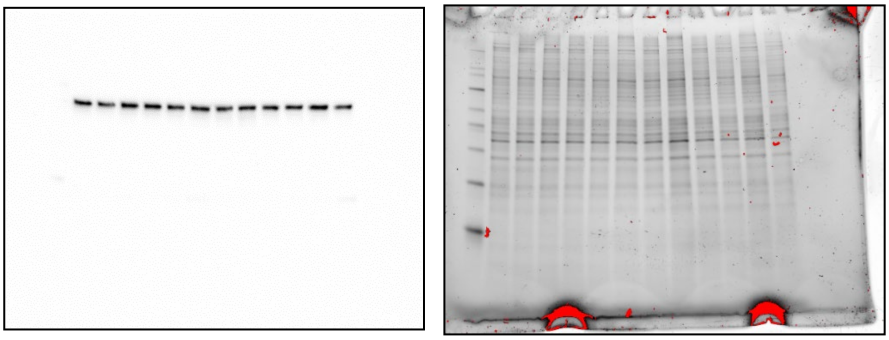

Figure S4: Full blot image and total protein gel for expression of E-Cadherin in response to mifepristone, in HaCaT, TYS, and SAS-H1 cells. **Key:** Lane 1: magic marker, lane 2: SFM 24 h, lane 3: EGF 24 h, lane 4: Mif 1μM 24 h, lane 5: Mif 5μM 24 h, lane 6: Mif 10 μM 24 h, lane 7: Mif 20 μM 24 h, lane 8: SFM 48 h, lane 9: EGF 48 h, lane 10: Mif 1μM 48 h, lane 11: Mif 5μM 48 h, lane 12: Mif 10μM 48 h, and lane 13: Mif 20μM 48 h.
